# Development and validation of a diagnostic model for the identification of chronic ocular graft-versus-host disease (oGVHD)

**DOI:** 10.3389/fmed.2023.1277194

**Published:** 2023-10-27

**Authors:** Zhan Shen, Bohao Hu, Liyuan Tao, Jiao Ma, Rongmei Peng, Yinghan Zhao, Jing Hong

**Affiliations:** ^1^Department of Ophthalmology, Peking University Third Hospital, Beijing, China; ^2^Beijing Key Laboratory of Restoration of Damaged Ocular Nerve, Peking University Third Hospital, Beijing, China; ^3^Research Center of Clinical Epidemiology, Peking University Third Hospital, Beijing, China

**Keywords:** Chronic Ocular Graft-Versus-Host Disease, corneal fluorescein staining, diagnostic model, nomogram, Schirmer’s tear test

## Abstract

**Purpose:**

To verify the International Chronic Ocular Graft-Versus-Host Disease (ICCGVHD) Group diagnostic criteria and establish an easy-to-use and reliable diagnosis model for quick identification of chronic oGVHD.

**Methods:**

This study included 180 patients (355 eyes) who underwent allogeneic hematopoietic stem cell transplantation (allo-HSCT) and visited the Peking University Third Hospital Cornea and Ocular Surface Disease Specialist Clinic from July 2020 to February 2021. The proportion of chronic oGVHD was 76.06% (279/355).

**Results:**

Five complaints, including eye dryness, photophobia, foreign body sensation, eye redness, and burning sensation; six ophthalmic examinations, including Ocular Surface Disease Index (OSDI) score, corneal fluorescein staining (CFS), tear break-up time (TBUT), Schirmer’s test score without anesthesia, conjunctival score, tear meniscus height, and non-ocular GVHD-involved organs were significantly different between patients with chronic oGVHD and control group (*p* < 0.05). Binary logistic regression (backward LR algorithm) selection demonstrated that three variables retained diagnostic significance for chronic oGVHD: CFS (OR = 2.71 (1.92–3.81), *p* < 0.001), Schirmer’s test score without anesthesia (OR = 0.83 (0.76–0.91), *p* < 0.001), and conjunctival score (OR = 1.96 (1.13–3.42), *p* = 0.031). A nomogram for the identification of chronic oGVHD was developed, and its performance was examined using an internal validation cohort (118 eyes). The areas under the curve (AUCs) for the three-variable-based nomogram were 0.976 (95% CI (0.959–0.992), *p* < 0.01) and 0.945 (95% CI (0.904–0.986), *p* < 0.01) in the development and internal validation cohorts, respectively.

**Conclusion:**

This concise three-variable-based nomogram based on ICCGVHD criteria could serve as an easy-to-use and reliable tool for rapid screening of chronic oGVHD.

## Introduction

Allogeneic hematopoietic stem cell transplantation (allo-HSCT) has been generally accepted as the ultimate treatment for malignant hematologic diseases, aplastic anemia, mucopolysaccharidosis, lysosomal storage diseases and other metabolic diseases (1, 2). After more than 20 years of development, an increasing number of patients have received allo-HSCT and benefited from it. There are more than 20,000 HSCTs performed in the United States each year. With the improvement of the patient survival rate and survival time, many complications after transplantation have gradually been discovered and recognized (3, 4).

Graft-versus-host disease (GVHD) is the main complication of allo-HSCT, which occurs in 30–70% of post-HSCT patients. In addition, 60–90% of GVHD patients experience eye involvement (5–7). When ocular GVHD (oGVHD) occurs, a large number of inflammatory cells and inflammatory factors infiltrate the ocular surface tissues, such as the meibomian glands, lacrimal glands, conjunctiva, and cornea, causing acute and chronic inflammation, which can cause a large amount of normal tissue necrosis and apoptosis in a short time. The quality and quantity of tear fluid and the stability of tear film are all seriously affected. If not treated in time, it may cause severe pain and vision loss significantly reducing patients’ quality of life (QOL) (8–13).

There are currently two diagnostic metrics of oGVHD. The 2014 National Institutes of Health (NIH) criteria define a symptom-based diagnosis of oGVHD (8, 9), where oGVHD is the new onset of dry, gritty, or painful eyes with decreased values in the Schirmer’s test without anesthesia in a patient after allogeneic HSCT. This criteria is more concise and facilitates the determination of oGVHD by transplant clinicians.

In 2013, the International Chronic Ocular GVHD Consensus Group (ICOGCG) proposed new diagnostic metrics to increase objectivity in the diagnosis and follow-up of chronic GVHD (5). The ICOGCG identified four subjective and objective variables to measure in patients following HSCT: Ocular Surface Disease Index (OSDI) score, Schirmer’s score without anesthesia, corneal fluorescein staining (CFS) and conjunctival injection. Each variable is scored 0–2 or 0–3, with a maximum composite score of 11. Taking the presence or absence of systemic GVHD into consideration as well, patients are eventually assigned to one of three diagnostic categories: no, probable, or definite oGVHD. The ICCGVHD diagnostic criteria considered multiple clinical test parameters, were noted to be better at differentiating oGVHD patients from dry eye disease (DED). In 2022, Yoko Ogawa validated the ICCGVHD criteria. Good sensitivity, specificity, predictive value and correlation were found between ICCGVHD and NIH2014 (10).

There are also other diagnostic criteria, which have been used in studies on oGHVD. An extension of the Tear Film and Ocular Surface Society Dry Eye Workshop II (TFOS DEWS II) criteria required ocular surface discomfort symptoms with OSDI score ≥ 13 along with any one of the following: TFBUT <10 s; tear osmolarity >308 mOsm/L in either eye (or an inter-eye difference > 8 mOsm/L); ocular surface staining (>5 corneal spots, >9 conjunctival spots or lid wiper epitheliopathy of ≥2 mm in length and/or ≥ 25% sagittal width) (11). The Japanese Dry Eye Society criteria for diagnosing dry eye with a greater focus on unstable tear film (tear film breakup time [TFBUT <5 s]) and subjective symptoms (12).

The different sets of criteria present great subjectivity and variability in the best clinical practices (BCPs), diagnosis, staging, and treatment of chronic oGVHD. Which indicators exceed what criteria for a more effective diagnosis of chronic oGVHD and what are the weights? The diversity and complexity of diagnostic criteria make it difficult for some community ophthalmologists and hematologist to become familiar with and accurately recognize chronic oGVHD. However, the burden of treating more life-threatening complications often prevents patients from visiting specialty ophthalmology clinics for routine examinations, which may lead to misdiagnosis and delay of treatment of chronic oGVHD. Therefore, we hope to summarize an easy-to-use and reliable diagnostic method to screen chronic oGVHD. It can be mastered by most ophthalmologists and hematologist to recognize chronic oGVHD rapidly. In this way, more patients can still receive accurate diagnoses and timely management when they cannot visit specialty ophthalmology clinics for routine examinations.

According to the diagnostic list above, we choose all six ophthalmic examination variables, five subjective complaints, and non-ocular GVHD-involved organs into consideration. The purpose of this study was to determine which of the above indicators are predictive of oGVHD diagnosis, verify the ICCGVHD criteria and establish a practical and reliable tool based on the ICCGVHD criteria for the rapidly identification of chronic oGVHD, minimizing the subjectivity and variability of clinical diagnosis.

## Materials and methods

### Data sources and characteristics

In this study, we aimed to establish and validate a simple and practical tool for the early identification of chronic oGVHD in China by using onset symptoms and simple ophthalmic examination. This study was approved by the ethics committee of the Peking University Third Hospital. A total of 233 patients (413 eyes) who visited the Peking University Third Hospital Cornea and Ocular Surface Disease Specialist Clinic after HSCT were enrolled in our study from April 2021 to November 2021. To test the generalization of our model, we split the general cohort according to the order of patients’ visit times and used 67% for model training and 33% for internal model validation; we ensured a balanced data distribution.

Patients were excluded according to the following criteria: (1) signs of allergy, infection, glaucoma, retinopathy, or other immune diseases, (2) lack of complete medical records, and (3) inability to be followed up and interviewed in the clinic. The study was approved by the Peking University Third Hospital Medical Science Research Ethics Committee (protocol number: M20200489) and conducted in accordance with the Helsinki Declaration, and all participants in this study provided written consent (7, 13).

### Demographic variables collected for the study

Patient characteristics included demographics, type and reason for the transplant, HLA compatibility match, and non-ocular GVHD organ involvement. The general ocular conditions included (1) intraocular pressure (measured with a noncontact tonometer) and (2) best observed vision acuity (measured in logMAR). The patients complained of symptoms including eye dryness, eye redness, photophobia, lacrimation, foreign body sensation, and burning sensation, which were graded by the patients themselves. Higher scores represent more severe symptoms, with scores ranging from 0 to 5. We also used the OSDI to assess the subjective ocular symptoms of dry eye symptoms. The total OSDI was calculated using the following formula and ranges from 0 to 100: OSDI = (sum of scores for all questions answered × 100)/(total number of answered questions × 4) (14).

Objective ocular examinations included the following: (1) TBUT (15), (2) CFS score (16) (according to the National Eye Institute (NEI) grading scale, the examiner divided the cornea into five quadrants; no corneal epithelium staining in each quadrant scored 0; 1–10 stained points scored 1; 10–30 stained points scored 2; and > 30 stained points without fusion and/or corneal filiform paraphyte and/or punctate keratopathy fusion and/or corneal ulcer scored 3; the sum of the score in each quadrant was the final result for each eye), (3) Schirmer’s test score without anesthesia, (4) lower tear meniscus height, and (5) conjunctival hyperemia score (17) (Scored from 0 to 4 according to the Institute for Eye Research-Brien Holden Vision Institute scales:0 indicated an absence of conjunctival hyperemia; 1, very slight conjunctival hyperemia; 2, slight conjunctival hyperemia; 3, moderate conjunctival hyperemia; and 4, severe conjunctival hyperemia).

### Outcomes

We defined the occurrence of chronic oGVHD on the basis that patients’ ocular and systemic manifestations and referred to both the 2014 NIH Chronic GVHD diagnostic criteria and the ICCGVHD criteria for chronic oGVHD. Diagnoses were confirmed independently by three experts in our center (5, 18).

### Statistical methods and model establishment

Univariate factor logistic regression was used in the model training group to analyze and calculate the related factors of chronic oGVHD. Three features with statistically significant odds ratios (ORs) were identified through binary logistic regression (LR) (backward LR method). On this basis, we established a nomogram based on the variables selected from the model.

The accuracy of the chronic oGVHD diagnostic score was assessed using the area under the receiver operator characteristic curve (AUC). We also used the calibration curve to validate the generalizability of the chronic oGVHD diagnostic score. Statistical tests were done with R software (version 3.6.0) and SPSS (version 22.0). Statistical significance was set at two-sided *p* values less than 0.05.

## Results

**Figure 1:**
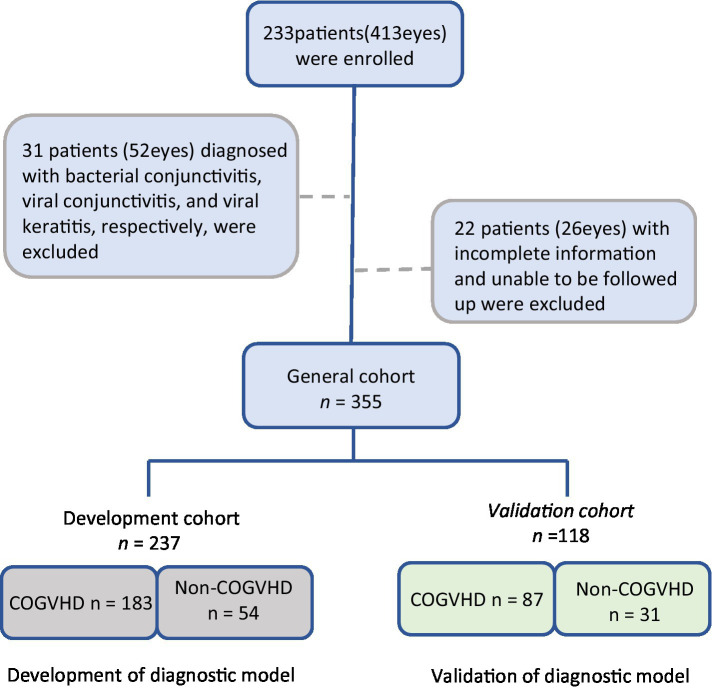
Flow diagram of patient selection, COGVHD, chronic ocular graft-versus-host-disease.

Two hundred and thirty-three oGVHD patients (413 eyes) were initially recruited, among which 31 patients (52 eyes) were excluded for other ocular diseases and 22 patients (26 eyes) were complete medical records. Eventually, a total of 180 patients (355eyes) who underwent HSCT were enrolled in our study (Figure 1). The median age of the patients was 55.53 years (IQR: 37, 63); The demographic and clinical characteristics of the study population are presented in Table 1.

**Table 1:** Demographic and clinical characteristics of patients after allo-HSCT.

Variable	Post-HSCT			^a^*p* values
	Total sample (*N* = 180)	Modeling (*N* = 120)	Validation (*N* = 60)	
Age (SD)	30 ± 16	31 ± 15	28 ± 16	*p* = 0.230
Sex, *n* (%)				*p* = 0.526
Female	84 (46.6%)	54 (45.0%)	30 (50%)	
Male	96 (53.4%)	66 (55.0%)	30 (50%)	
Sex relation patient/donor, *n* (%)				*p* = 0.375
Female/female (nonpregnant)	6 (3.3%)	4 (3.3%)	2 (3.3%)	
Female/female (pregnant)	29 (16.1%)	20 (16.6%)	9 (15%)	
Female/male	46 (25.5%)	30 (25%)	16 (26.7%)	
Male/female (nonpregnant)	10 (5.6%)	8 (6.7%)	2 (3.3%)	
Male/female (pregnant)	28 (15.6%)	23 (19.2%)	5 (8.3%)	
Male/male	51 (28.3%)	30 (25%)	21 (35%)	
Not available	10 (5.6%)	5 (4.2%)	5 (8.3%)	
Type of transplant, *n* (%)				*p* = 0.091
Matched (10/10 or 9/10)	69 (38.3%)	52 (43.3%)	17 (28.4%)	
Mid Matched (5/10)	103 (57.2%)	65 (54.2%)	38 (66.3%)	
Not available	8 (4.4%)	3 (2.5%)	5 (8.3%)	
Primary blood disease				*p* = 0.055
ALL	45 (25%)	26 (21.7%)	19 (31.7%)	
AML	71 (39.4%)	48 (4%)	23 (38.3%)	
CML	8 (4.4%)	5 (4.2%)	3 (5%)	
Lymphoma	5 (2.8%)	3 (2.5%)	2 (3.3%)	
MDS	31 (17.2%)	26 (21.7%)	5 (8.3%)	
Hemophagocytic syndrome	1 (0.6%)	0	1 (1.7%)	
AAA	8 (4.4%)	8 (6.7%)	0	
Not available	11 (6.1%)	4 (3.3%)	7 (11.7%)	
IOP (SD)		14.73 ± 5.33	15.21 ± 4.45	*p* = 0.378
BCVA, log MAR (SD)		0.31 ± 0.31	0.30 ± 0.31	*p* = 0.795

Allo-HSCT, allogeneic hematopoietic stem cell transplantation; ALL, acute lymphoblastic leukemia; AML, acute myeloid leukemia; CLL, chronic lymphocytic leukemia; CML, chronic myeloid leukemia; MM, multiple myeloma, MDS, myelodysplastic syndrome; AAA, acute aplastic anemia.

a *p* value < 0.05 was considered statistically significant.

The patients were divided into development and validation cohorts, according to the order of visit dates. The characteristics of the patients in the development cohort (n = 237) and internal validation cohort (*n* = 118) were similar (Table 1).

**Figure 2:**
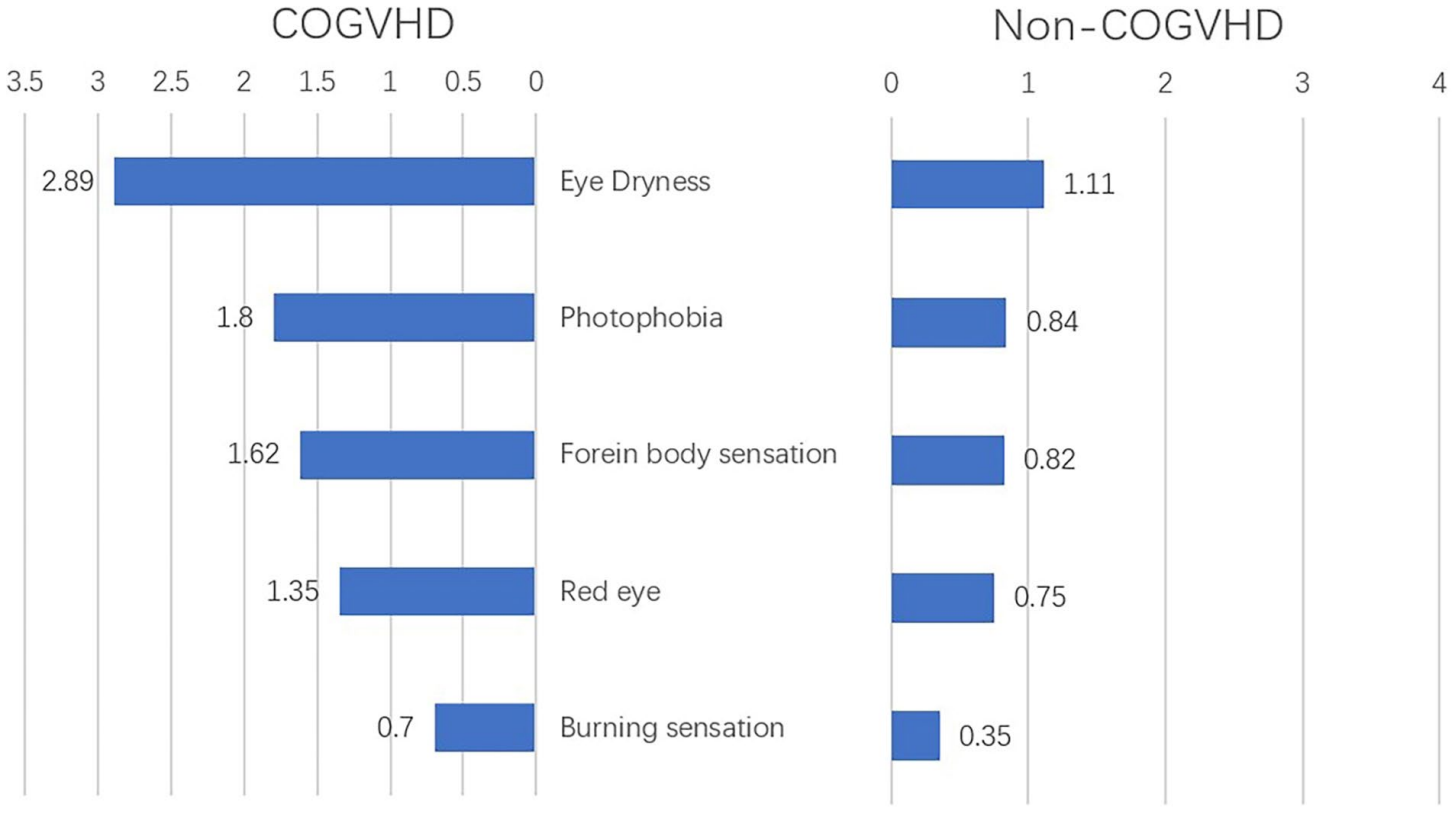
Complains at onset of illness in chronic ocular GVHD and non-chronic ocular GVHD cases (*n* = 237); COGVHD, chronic ocular graft-versus-host-disease.

In the development cohort, 183 of 237 (77.2%) eyes were diagnosed with chronic oGVHD. Compared with those with non-chronic oGVHD, patients diagnosed with chronic oGVHD had higher average scores of eye dryness (2.89 vs. 1.11), photophobia (1.8 vs. 0.84), foreign body sensation (1.62 vs. 0.82), eye redness (1.35 vs. 0.75) and burning sensation (0.7 vs. 0.35) (Figure 2). The results of univariate logistic regression analysis showed that five complaints above and seven objective examinations, including OSDI score, CFS, TBUT, Schirmer’s test score without anesthesia, conjunctival score, tear meniscus height and non-ocular involvement organs may be significant diagnostic factors for chronic oGVHD (all *p* < 0.05, Supplementary Table S1).

In the multivariable logistic regression model, considering the collinearity between some variables, ten variables were identified as independent variables: photophobia, foreign body sensation, burning sensation, OSDI score, CFS, TBUT, Schirmer’s test, conjunctival score, lacrimal meniscus height and average no. affected organs. After binary logistic regression (backward LR algorithm), three variables retained diagnostic significance for chronic oGVHD: CFS [OR = 2.71 (1.92–3.81), *p* < 0.001], Schirmer’s test score without anesthesia [OR = 0.83 (0.76–0.91), p < 0.001], and conjunctival score [OR = 1.96 (1.13–3.42), *p* = 0.031] (Table 2).

**Table 2:** Diagnostic factors for chronic ocular GVHD in the development cohort: univariable and multivariable models.

	cOR (95% CI)	^a^*p* values	aOR (95% CI)	^a^*p* values
Photophobia	2.24 (1.59–3.16)	<0.001	–	–
Foreinbody sensation	1.55 (1.17–2.06)	0.002	–	–
Burning sensation	1.62 (1.04–2.52)	0.033	–	–
OSDI	1.03 (1.02–1.05)	<0.001	–	–
CFS	12.35 (4.58–33.12)	<0.001	2.71 (1.92–3.81)	<0.001
TBUT	0.69 (0.62–0.77)	<0.001	–	–
Schirmer’s test (mm)	0.70 (0.63–0.79)	<0.001	0.83 (0.76–0.91)	<0.001
Conj	4.71 (2.19–10.15)	<0.001	1.96 (1.13–3.42)	0.031
Tear Meniscus	0.21 (0.04–1.26)	0.09	–	–
Average no. affected organs	1.48 (1.06–2.07)	0.021	–	–

cOR, crude odds ratio; aOR, adjusted odds ratio; CFS, corneal fluorescein staining; Conj, conjunctival score; OSDI, Ocular Surface Disease Index.

a *p* value < 0.05 was considered statistically significant.

In the multivariable model, the significant variables were selected using a backward LR procedure from three complains including photophobia, foreign body sensation, burning sensation and six objective ocular examinations including OSDI score, CFS, TBUT, Schirmer’s test score without anesthesia, conjunctival score, tear meniscus.

**Figure 3:**
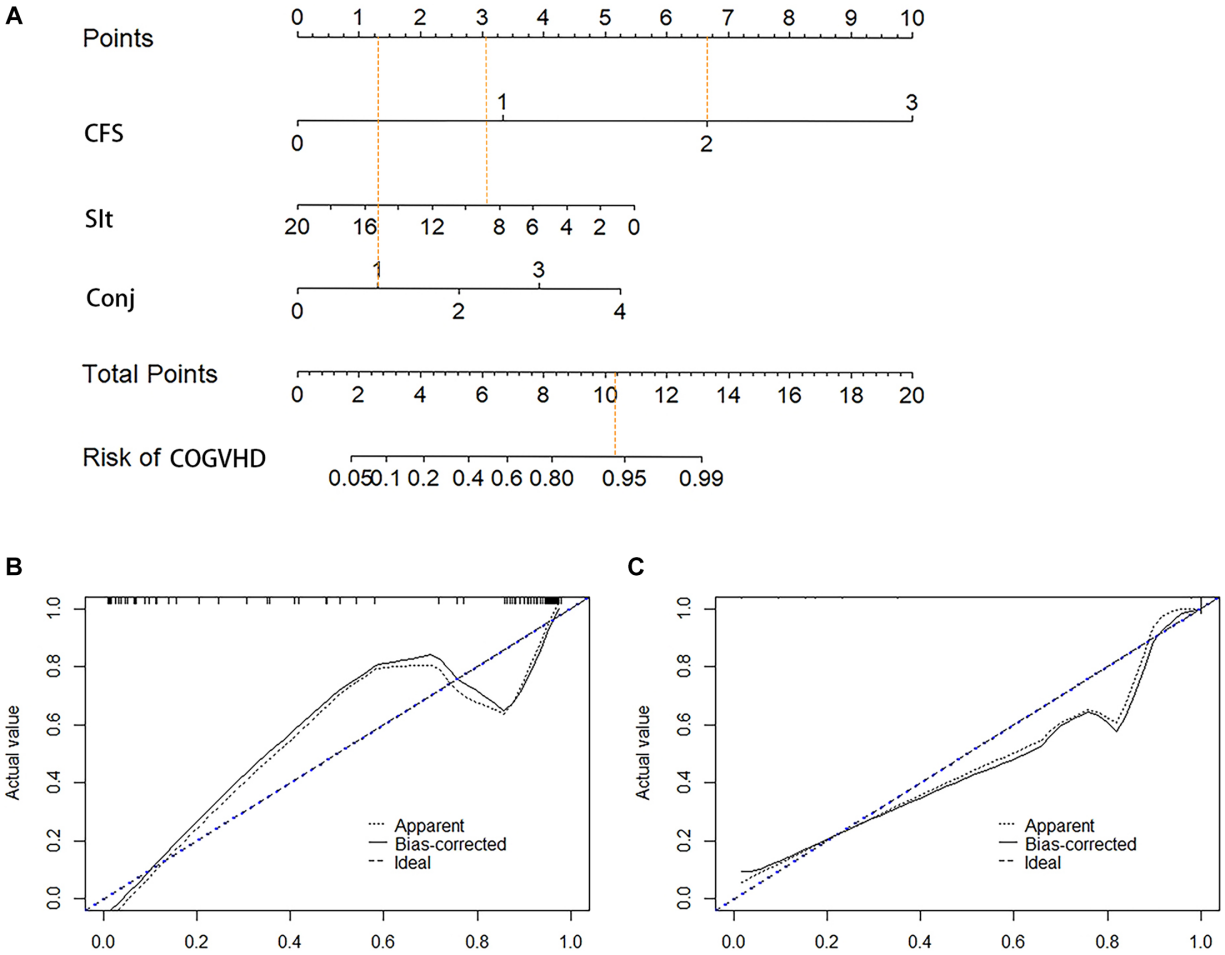
Nomogram and calibration curves for diagnosis chronic ocular GVHD. **(A)** Nomogram model for diagnosis chronic ocular GVHD. **(B)** Calibration curves for the nomogram in the development cohort. **(C)** Calibration curves for the nomogram in the validation cohort; CFS, corneal fluorescein staining; Sit, Schirmer’s I test (mm); Conj, conjunctival score; COGVHD, chronic ocular graft versus host disease.

**Figure 4:**
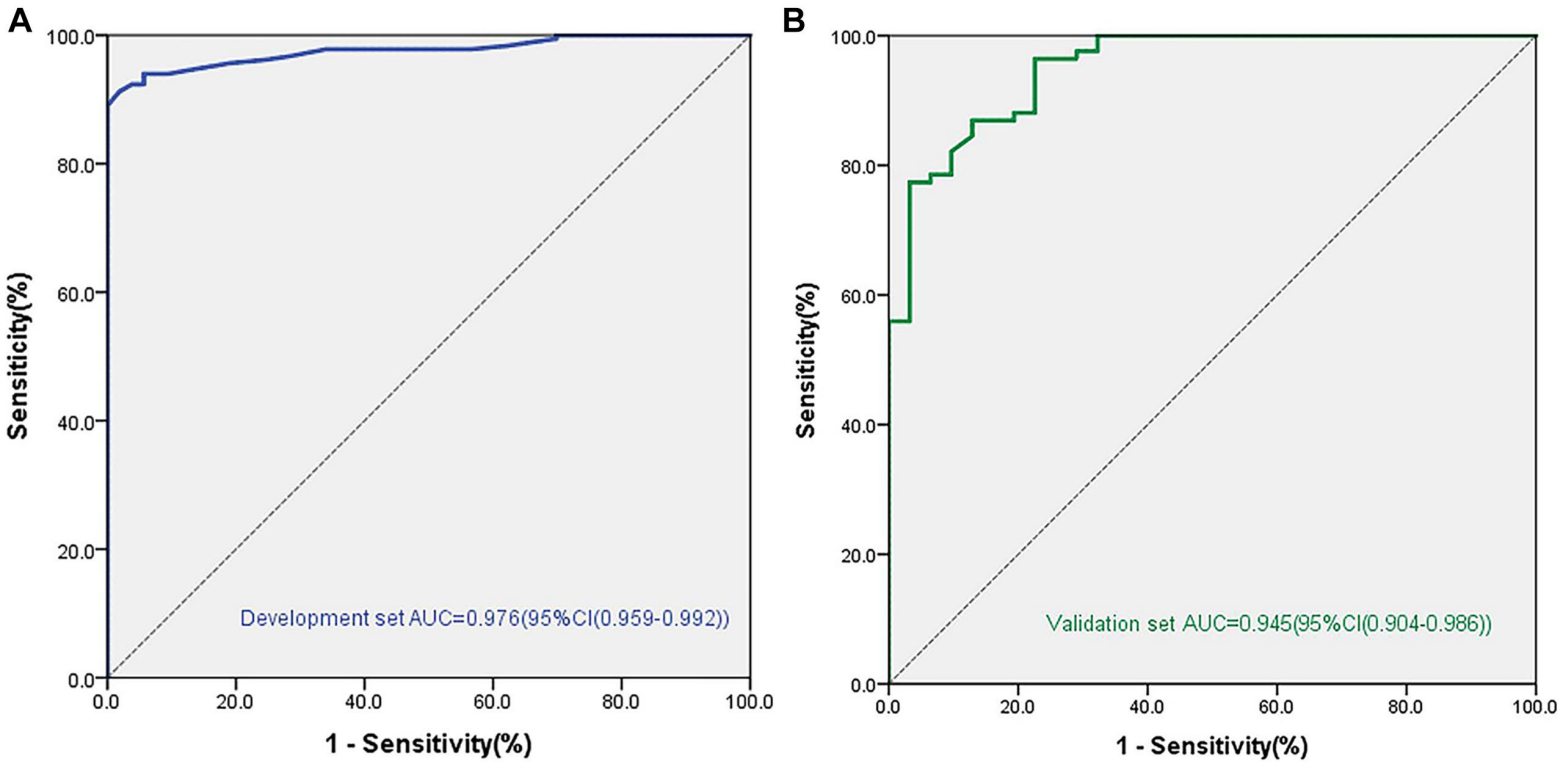
Areas under the curve (AUC) in the nomogram model for diagnosis chronic ocular GVHD in the development and validation cohorts. **(A)** AUC for the development cohorts. **(B)** AUC for the validation cohorts.

We established a nomogram based on the variables selected from the model (CSF, Schirmer’s test score and conjunctival score) to diagnose chronic oGVHD (Figure 3). In the development cohort, the AUC for the nomogram was 0.976 [95% CI (0.959, 0.992), *p* < 0.01] (Figure 4).

The validation cohort included 118 eyes. Eighty-seven of 118 (73.7%) eyes were diagnosed with chronic oGVHD. The AUC was 0.945 [95% CI (0.904, 0.986), *p* < 0.01], and the calibration curve also showed that the nomogram had good calibration (Figure 4).

## Discussion

OGVHD has at least three important biological processes: lacrimal gland dysfunction, meibomian gland dysfunction, and corneal and conjunctival inflammatory infiltration (19, 20, 21). The pathogenesis of oGVHD can currently be summarized as a three-phase model. The initial stage is considered to be an inflammatory process mediated by T cells, and the subsequent stage is the result of the immune cascade (13, 22). The host’s immune regulatory response is insufficient to control early inflammation. As a result, chronic inflammation and immune disorders occur, leading to changes in glandular fibrosis and ineffective tear film, leading to ocular surface damage (23). The medium-sized ducts of the lacrimal gland are preferentially targeted by T cells and other inflammatory cells in the initial stage (24). The ducts of the lacrimal and meibomian glands and the nasolacrimal duct are often blocked by immune-mediated fibrosis. Other areas that may be affected include the cornea, limbus, and conjunctiva. Confocal microscopy of patients with oGVHD showed that the infiltration of globular immune cells and dendritic cells around the basal nerve in the central cornea and limbus area increased, indicating the infiltration of active immune cells into the eye with GVHD in avascular corneal disease (25).

The current internationally recognized diagnostic criteria for chronic oGVHD are divided into the diagnostic criteria for chronic GVHD proposed by the NIH in 2005, which were improved in 2014, and the diagnostic scoring criteria proposed by the ICCGVHD in 2013 (26). The NIH’s diagnostic criteria are simple and easy to implement, and the NIH score combined with the Schirmer’s test shows >90% sensitivity and specificity for the diagnosis of oGVHD (27), but the diagnostic criteria are challenging for several reasons, including the limited understanding of the pathophysiology, the lack of validated measurement tools and scoring systems etc. Inamoto also demonstrated that the Schirmer’s test did not correlate well with the change in oGVHD severity (28). In past studies (29, 30), it has been suggested that the ICCGVHD diagnostic criteria have good consistency and repeatability, and perform better at differentiation oGVHD patients from non-oGVHD DED. There are numerous criteria for the diagnosis of ocular GVHD, with different criteria focusing on different indicators.

Based on the BCP of chronic oGVHD, our study diagnosed 180 patients 355 eyes after allo-HSCT, divided the eyes into a disease group and a non-disease group, and evaluated the clinical manifestations and clinical examination parameters of the patients.

Our results showed that eye dryness, photophobia, foreign body sensation, eye redness and burning sensation were significantly different between the patient group and the control group. The characteristics are generally similar to those mentioned in Shikari’s study, with slight differences (burning, irritation, pain, redness, blurry vision, foreign body sensation, and photophobia) (31). There were also significant differences in OSDI score, CFS, TBUT, Schirmer’s test score without anesthesia, conjunctival score and tear meniscus height. This result confirms that the clinical manifestations of patients with oGVHD are a reliable indicator for the diagnosis of chronic oGVHD, which is consistent with the diagnostic criteria of the NIH. Simultaneously, we also verified that OSDI score, CFS, TBUT, Schirmer’s test score without anesthesia, conjunctival score and tear meniscus height were statistically significant, further verifying the ICCGVHD diagnostic criteria.

We then selected these statistically significant indicators for further analysis. According to the results of logistic regression analysis, we ultimately found three indicators with significant differences between the two groups, including CFS [OR = 2.71 (1.92–3.81), *p* < 0.001], Schirmer’s test score without anesthesia [OR = 0.83 (0.76–0.91), *p* < 0.001], and conjunctival score [OR = 1.96 (1.13–3.42), *p* = 0.031]. In the development cohort, the AUC for the nomogram was 0.976 [95% CI (0.959, 0.992), *p* < 0.01]. We verified this model in another 118 patients, and the AUC was 0.945 [95% CI (0.904, 0.986), *p* < 0.01], which demonstrated that the model has good repeatability and consistency.

A nomogram was established based on the variables selected from the model (CFS, Schirmer’s test score and conjunctival score) to diagnose chronic oGVHD (Figure 3). According to the nomogram, CFS and Schirmer’s test are the most effective in diagnosing chronic oGVHD. This result further validates the ICCGVHD diagnostic criteria and simplifies the diagnostic criteria. We also found that when CFS was greater than 3 points, almost all cases were diagnosed as chronic oGVHD. When CSF was less than 3 points, the conjunctival score and Schirmer’s test were needed to calculate the total score. When the total score was greater than 11, the probability of being diagnosed with chronic oGVHD was as high as 95%.

In summary, our research results indirectly reflected the sensitivity and specificity of the 2014 NIH diagnostic criteria and the 2013 ICCGVHD diagnostic criteria, and through further analysis of clinical symptoms and clinical parameters, a more simplified diagnostic model was obtained. Compared to NIH, this diagnostic model adds objective ocular examination scoring systems. But more concise than the ICCGVHD diagnostic criteria, facilitates the determination of chronic oGVHD by transplant clinicians and community ophthalmologists.

This diagnostic model was proven to have good sensitivity and specificity to help ophthalmologists and hematologist rapidly diagnose chronic oGVHD. However, there are still some limits in this study. A large-scale multicenter study is needed to verify the diagnostic model determine the best combination of clinical indicators to maximize the diagnostic sensitivity and specificity and determine the severity of chronic oGVHD, which will be the direction of our subsequent research.

## Conclusion

Our research results indirectly reflected the sensitivity and specificity of the 2014 NIH diagnostic criteria and the 2013 ICCGVHD diagnostic criteria, and through further analysis of clinical symptoms and clinical parameters, a more concise diagnostic model based on ICCGVHD criteria was obtained. This diagnostic model was proven to have good sensitivity and specificity to help ophthalmologists and hematologists rapidly diagnose chronic ocular GVHD.

## Data availability statement

The raw data supporting the conclusions of this article will be made available by the authors, without undue reservation.

## Ethics statement

The studies involving humans were approved by Peking University Third Hospital Institutional Review Board. The studies were conducted in accordance with the local legislation and institutional requirements. The participants provided their written informed consent to participate in this study.

## Author contributions

ZS: Conceptualization, Data curation, Methodology, Writing – original draft, Formal analysis, Investigation. BH: Methodology, Writing – original draft. LT: Conceptualization, Data curation, Investigation, Methodology, Writing – original draft. JM: Investigation, Supervision, Writing – review & editing. RP: Investigation, Supervision, Writing – review & editing. YZ: Investigation, Writing – review & editing. JH: Conceptualization, Formal analysis, Methodology, Project administration, Supervision, Writing – review & editing.

## Figures and Tables

**Table:** 

allo-HSCT	allogeneic hematopoietic stem cell transplantation
AUCs	areas under the curve
BCPs	best clinical practices
CFS	corneal fluorescein staining
CML	chronic myeloid leukemia
DED	dry eye disease
GVHD	Graft-versus-host disease
ICOGCG	International Chronic Ocular GVHD Consensus Group
LR	logistic regression
NEI	National Eye Institute
NIH	National Institutes of Health
oGVHD	ocular graft-versus-host disease
ORs	odds ratios
OSDI	Ocular Surface Disease Index
QOL	quality of life
TBUT	tear break-up time
TFOS DEWS II	Tear Film and Ocular Surface Society Dry Eye Workshop II
