# Supplementary material for: Development and validation of a diagnostic model for the identification of chronic ocular graft-versus-host disease (oGVHD)
Source: Front Med (Lausanne). 2023 Oct 27;10:1277194. doi: 10.3389/fmed.2023.1277194 (PMC10641834; doi:10.3389/fmed.2023.1277194)
Supplement: Supplementary file 1 [file Table_1.DOCX]

**Supplement table 1.** Complains and objective examinations of patients with or without chronic ocular GVHD.

|  | COGVHD | WITHOUT COGVHD | ^a^ P values |
| --- | --- | --- | --- |
| Eye dryness | 1.10±1.32 | 3.00±1.10 | ＜0.001 |
| Photophobia | 0.70±0.93 | 1.73±1.33 | ＜0.001 |
| Foreign  body sensation | 0.90±1.12 | 1.58±1.37 | 0.002 |
| Eye redness | 0.79±0.93 | 1.32±1.08 | 0.002 |
| Burning sensation | 0.37±0.76 | 0.70±0.97 | 0.029 |
| OSDI | 22.78±20.27 | 36.68±22.84 | ＜0.001 |
| CFS | 0.09±0.30 | 7.07±4.82 | ＜0.001 |
| TBUT | 6.14±3.00 | 2.79±2.42 | ＜0.001 |
| Schirmer's  test (mm) | 9.55±7.36 | 2.48±2.39 | ＜0.001 |
| Conj | 0.13±0.63 | 0.70±0.83 | ＜0.001 |
| Tear Meniscus | 0.24±0.10 | 0.15±0.27 | 0.013 |
| Average no. affected organs | 2±2 | 2±1 | 0.015 |

COGVHD = chronic ocular graft versus host disease; OSDI = Ocular Surface Disease Index; CFS = corneal fluorescein staining; TBUT = tear break-up time; Conj = conjunctival injection;

^a^ P value <0.05 was considered statistically significan.
